# Insight of Saffron Proteome by Gel-Electrophoresis

**DOI:** 10.3390/molecules21020167

**Published:** 2016-01-29

**Authors:** Gianluca Paredi, Samanta Raboni, Francesco Marchesani, Stella A. Ordoudi, Maria Z. Tsimidou, Andrea Mozzarelli

**Affiliations:** 1 Department of Pharmacy, Interdepartment Center SITEIA.PARMA, University of Parma, Parma 43124, Italy; gianluca.paredi@unipr.it (G.P.); samanta.raboni@unipr.it (S.R.); francesco.marchesani@studenti.unipr.it (F.M.); 2 Laboratory of Food Chemistry and Technology, School of Chemistry, Aristotle University of Thessaloniki, Thessaloniki 54124, Greece; steord@chem.auth.gr (S.A.O.); tsimidou@chem.auth.gr (M.Z.T.); 3 National Institute of Biostructures and Biosystems, Rome 00136, Italy; 4 Institute of Biophysics, CNR, Pisa 56124, Italy

**Keywords:** saffron, *Crocus sativus* L., proteomics, adulteration, *Gardenia jasminoides*, *Carthamus tinctorius* L.

## Abstract

Saffron is a spice comprised of the dried stigmas and styles of *Crocus sativus* L. flowers and, since it is very expensive, it is frequently adulterated. So far, proteomic tools have never been applied to characterize the proteome of saffron or identify possible cases of fraud. In this study, 1D-Gel Electrophoresis was carried out to characterize the protein profile of (i) fresh stigmas and styles of the plant; (ii) dried stigmas and styles from different geographical origins (Spanish, Italian, Greek and Iranian) that had been stored for various periods of time after their processing; and (iii) two common plant adulterants, dried petals of *Carthamus tinctorius* L. and dried fruits of *Gardenia jasminoides* Ellis. A selective protein extraction protocol was applied to avoid interference from colored saffron metabolites, such as crocins, during electrophoretic analyses of saffron. We succeeded in separating and assigning the molecular weights to more than 20 proteins. In spite of the unavailability of the genome of saffron, we were able to identify five proteins by Peptide Mass Fingerprinting: phosphoenolpyruvate carboxylase 3, heat shock cognate 70 KDa protein, crocetin glucosyltransferase 2, α-1,4-glucan-protein synthase and glyceraldehydes-3-phosphate dehydrogenase-2. Our findings indicate that (i) few bands are present in all saffron samples independently of origin and storage time, with amounts that significantly vary among samples and (ii) aging during saffron storage is associated with a reduction in the number of detectable bands, suggesting that proteases are still active. The protein pattern of saffron was quite distinct from those of two common adulterants, such as the dried petals of *Carthamus tinctorius* and the dried fruits of *Gardenia jasminoides* indicating that proteomic analyses could be exploited for detecting possible frauds.

## 1. Introduction

Saffron is a spice comprised of the dried stigmas and styles of *Crocus sativus* L., a perennial plant belonging to the Iridaceae family*. Crocus sativus* L. is a sterile triploid species with the complete genome not yet being determined. However, several investigations are ongoing towards this goal [1,2,3,4] as well as studies aimed at unveiling pathways leading to the formation of key metabolites [5].

Saffron is presently produced in Asia (Iran, India, Afghanistan), Europe (Greece, Spain, Italy, France, Austria), and Africa (Morocco). The spice is derived from the three-branch styles of the *C. sativus* L. flowers after drying. The latter procedure is carried out in various ways, depending on the country and local traditional practices, typically for Spanish saffron at 70 °C for half an hour, for Italian saffron at 45 °C for several hours and for Greek production at controlled room temperature for 12–24 h [6]. The drying causes a loss in weight of about 20%, protein degradation and chemical transformations. The resulting product contains more than 150 different chemical entities that are at the basis of the very appreciated culinary properties as well as the many claims of beneficial health effects, most of them related with *in vivo* antioxidant properties [7] including neuropathologies [8], cancer [9]*,* depression [10], cardiopathies [11] and macular degeneration [12].

Saffron quality is associated with crocins that provide the typical yellow color, picrocrocin that confers a bitter taste, and safranal that causes a characteristic aroma. Crocins are water-soluble and belong to the carotenoids [13,14,15]. The most abundant crocin is *trans*-di-(β-d-gentiobiosyl) crocetin, that contains two dicarboxylic acid moieties esterified with gentiobiose. Picrocrocin is a terpene, very soluble in water and in alcoholic solutions, formed after the oxidative degradation of zeaxanthin [15]. Safranal is generated during stigma drying due to the hydrolysis of picrocrocin and is the major volatile compound, not very soluble in water but more soluble in alcoholic solutions [16]. Since saffron is the most expensive spice in the world, many different frauds and adulterations have been detected, including dyes, flower fruits or petals, and even colored meat [17,18,19,20].

Proteomics aims at the mapping of proteins in biological samples. Proteomic approaches are used for a wide range of applications, from biomarker identification [21,22] to food quality evaluation [23,24]. Different methods are applied that are either gel-based or gel-free, consisting of mono and bidimensional electrophoresis coupled to mass spectrometry and LC-mass spectrometry. In the present work, the analytical power of proteomic analyses is exploited for the initial characterization of saffron protein profile. Previous proteomics investigations of saffron were aimed at detecting proteins that bind crocins [25] and proteins associated with saffron embryogenic and non-embryogenic calli [26]. Here, we specifically compared (i) the proteome of fresh stigmas and styles with those of dried ones and stored for different periods of time; (ii) the proteome of saffron obtained from Spain, Italy, Greece and Iran; and (iii) the proteome of saffron with respect to that of *Gardenia jasminoides* fruits and *Carthamus tinctorius* L. petals, two commonly used adulterants. Proteins contained in saffron were identified by peptide mass fingerprint analysis. Overall, the present study is the first step towards the development of a comprehensive saffron proteome database, integrated with other omic and chemical data.

## 2. Results and Discussion

To our knowledge, this study is the first attempt of exploiting proteomic tools for the complete determination of saffron proteome. However, 1D-PAGE gel analysis of saffron stigmas and styles is not straightforward as the analysis of other food products and biological fluids due to the presence of high amount of interfering metabolites, such as the colored crocins. The dyes and other metabolites severely interfere with protein migration, both in isoelectrofocusing and SDS-PAGE experiments. For this reason, a tailored procedure for protein extraction from saffron stigmas and styles was developed (see Experimental Design). Only 1D-SDS-PAGE analyses were reproducibly carried out. In spite of these limitations, significant results were obtained and are reported below, opening the ways to further investigations on saffron proteome.

### 2.1. Comparison of Protein Patterns for Fresh and Dried Stigmas and Styles of C. sativus L. flowers

**Figure 1:**
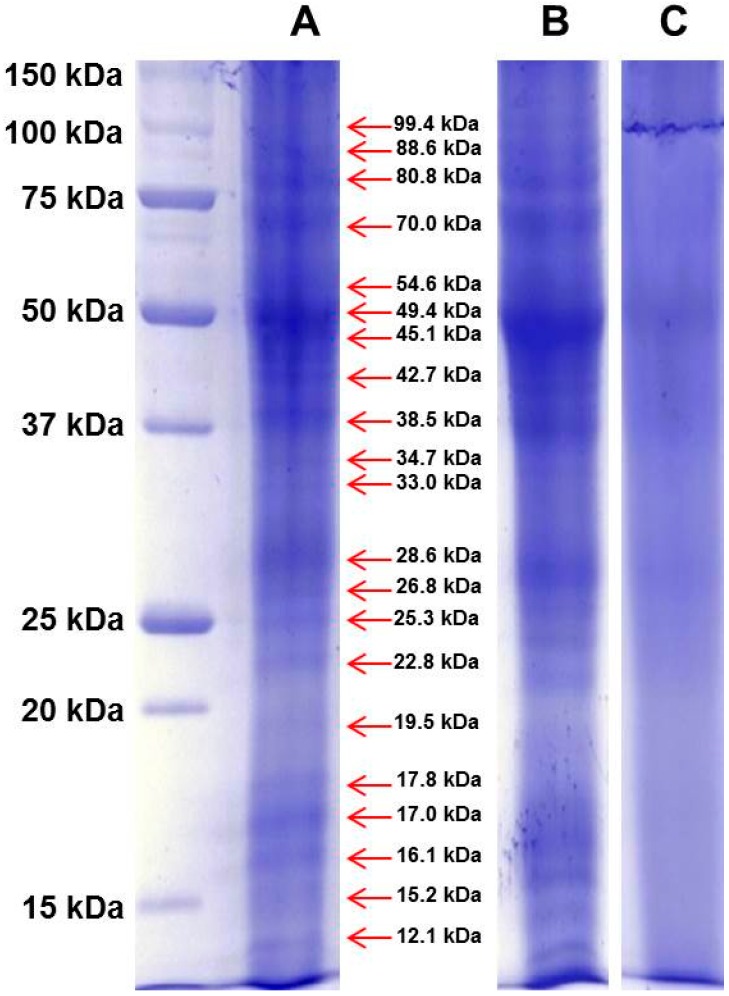
The proteins contained in Spanish saffron fresh stigmas and styles (**A**); dried stigmas and styles stored for two months (**B**); and dried stigmas and styles stored for three years (**C**) after processing were separated by 1D-SDS-PAGE, and stained with Bio-safe™ Coomassie. The first lane at left contains molecular weight markers. The red arrows indicate the molecular weight of identified bands of fresh stigmas and styles.

Proteins contained in fresh stigmas and styles of *Crocus sativus* L. flowers*,* and dried stigmas and styles, stored either for about two months or for more than three years after processing, were separated by 1D-SDS-PAGE (Figure 1). These materials were of the same geographical origin in Spain corresponding to the PDO product “Azafran de La Mancha”.

**Figure 2:**
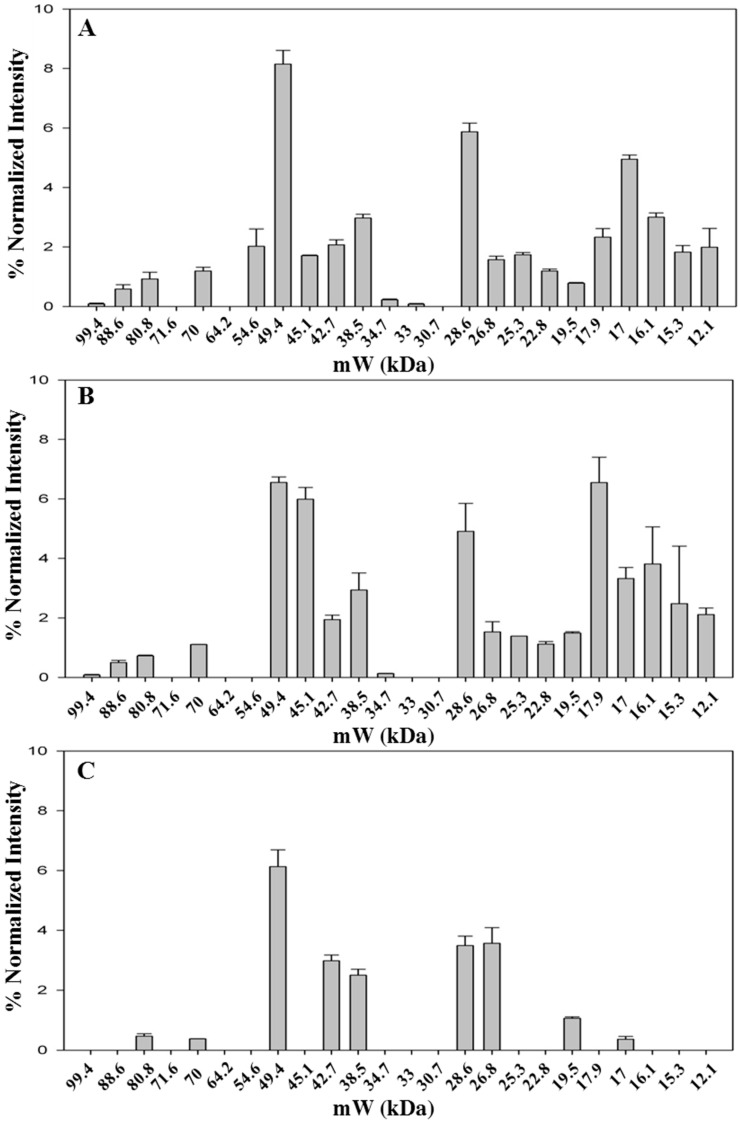
Bands detected in 1D-SDS-PAGE of fresh and styles stigmas (**A**); dried stigmas and styles stored for two months (**B**); and dried stigmas and styles stored for three years (**C**) after processing. Gel image was acquired with ChemiDoc™ MP System imager and analyzed with Image Lab™ software (version 5.2). Data are from two replicates.

As stated before, colored metabolites at high levels interfere with protein migration in 1D-PAGE resulting in a significant background noise. However, a robust gel image analysis, carried out with Image Lab™ software, allowed to identify several bands, as shown in a representative case in Figure S1. The pattern of identified bands is shown in Figure 2 (Table S1 shows the molecular weight of all bands identified in the saffron samples analyzed in this study).The fresh stigmas and styles contain a higher number of bands (*n* = 21) with respect to the dried ones (*n* = 19 and 10 for samples stored either for two months or for three years after processing, respectively). Even if the overall pattern is somewhat altered (Figure 2A,B), most bands are still detectable suggesting that drying by heating causes only a limited effect on protein precipitation and proteolysis. Nevertheless, the relative proportions among bands with molecular weight of 49.4, 45.1 and 17.9 are significantly modified after drying (Figure 2A,B and Figure S1).

For some bands (80.8 kDa, 70 kDa, 17 kDa), the relative intensity decreases with storage time (Figure S2), whereas for other bands, 26.8 kDa and 17.9 kDa, the relative intensity increases with time (Figure S2), possibly due to the overlapping with fragments generated by proteolysis of higher molecular weight proteins. The sample stored for three years after processing was found to contain a very low number of bands (Figure 2C), strongly suggesting that protease activity is ongoing during the storage of the spice. As a corollary, this finding indicates that other enzymes might also continue to be active, leading to a time-dependent change in saffron metabolite composition. This hypothesis is supported by a metabolomic study carried out exploiting the resolving power of NMR, that was able to discriminate saffron as a function of storage time after processing [27].

### 2.2. Identification of Proteins Contained in Fresh and Dried Stigmas and Styles

Among the bands that were detected in the sample of fresh stigmas and styles, fourteen were subjected to peptide mass fingerprint (PMF) analysis using a MALDI TOF/TOF mass spectrometer in order to identify the corresponding proteins. Saffron protein identification poses some problems because the genome of *Crocus sativus* L. stigmas and styles has not been determined yet and the EST sequence database is not accessible). We interrogated Swiss-prot *Viridiplantae* database using Mascot server (Matrix Science) as search engine. The resulting matches and coverages are reported in Table 1 whereas amino acid sequences of identified peptides are reported in Table S2. Only five proteins were identified with sufficient confidence. These are: (i) band at 99.4 kDa, present in very low quantity, identified as phosphoenolpyruvate carboxylase-3, an enzyme involved in glycogenesis; (ii) band at 70 kDa, identified as heat shock cognate 70 kDa protein, a protein that plays a crucial role in protecting plants against abiotic stress [28]; (iii) band at 49.4 kDa, present in large quantity in all analyzed samples, identified as crocetin glucosyltransferase-2, the enzyme that catalyzes the esterification of a carbonyl group of crocetin or crocetin monoglycosyl ester and formation of crocins, key components of saffron [29,30]; (iv) band at 42.7 kDa, identified as α-1,4-glucan-protein synthase, an enzyme involved in the synthesis of cell wall polysaccharide [31]; (v) band at 38.5 kDa, identified as glyceraldhyde-3-phosphate dehydrogenase-2, the enzyme carrying out the only oxidative step in glycolysis, but also involved in other moonlighting activities [32].

**Table 1:** Identity of proteins contained in the sample of fresh *C. sativus* L. stigmas and styles.

Band	Protein	Uniprot	Score ^a^	Coverage %
99.4 kDa	Phosphoenol piruvate carboxylase 3	CAPP3_ARATH	83	13
70 kDa	Heat shock cognate 70 kDa protein	HSP7C_PETHY	65	29
49.4 kDa	Crocetin glucosyl transferase-2	GLT2_CROSA	78	30
42.7 kDa	Α-1,4 glucan-protein synthase	UPTG_PEA	64	22
38.5 kDa	Glyceraldehyde-3-phosphate dehydrogenase-2 ^b^	G3PC_ORYSJ	85	34

^a^ Mascot score. All values are above the threshold of confidence; ^b^ Identification was further confirmed by MALDI MS/MS.

### 2.3. Comparison of Protein Pattern for Saffron from Different Origins

**Figure 3:**
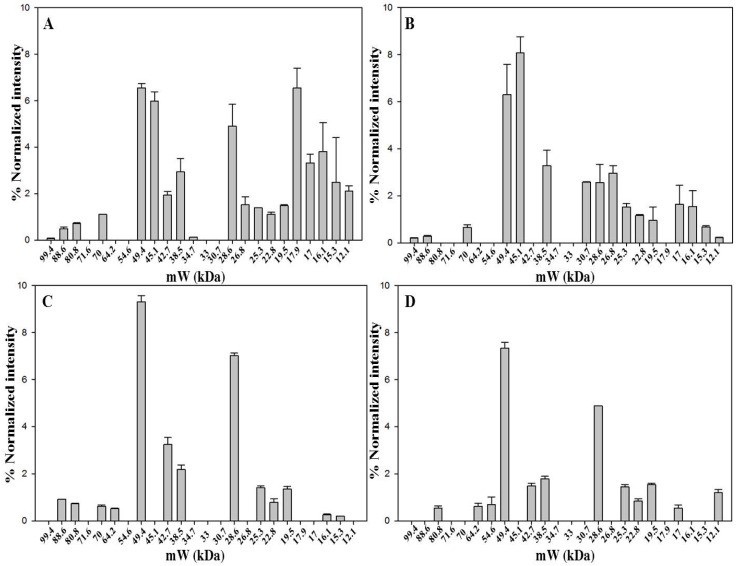
Bands detected in 1D-SDS-PAGE of saffron samples from Spain (**A**); Italy (**B**); Greece (**C**) and Iran (**D**). Gel image was acquired with ChemiDoc™ MP System imager and analyzed with Image Lab™ software (version 5.2). Data are from two replicates.

The aim was to characterize the protein pattern of saffron from different geographic origins, processed and stored in different ways, as another step towards the development of a saffron proteomic database. Proteins contained in dried stigmas and styles that were received from Spanish, Italian, Greek and Iranian producers were thus analyzed by 1D-SDS-PAGE (Figure 3 and Figure S3). The storage time after processing varied from two months (Spanish sample) to four months (Italian sample) and even three years (Greek and Iranian samples). Moreover, different geographical origin signifies different processing conditions e.g., drying procedure, 70 °C for the Spanish saffron, controlled room temperature for the Greek saffron, unknown for the Iranian saffron, and 45 °C for the Italian one. The heating time is expected to have an impact on the protein pattern of the produced saffron because it affects the degree of cell disruption and protease activity. Moreover, the storage time seems to play a major role in dictating the number of detected bands. The longer the storage time, the lower is the number of detected proteins. Not surprisingly, the band patterns of the examined saffron samples differed according to the number of detected proteins, which ranged from 19 for the Spanish and 16 for the Italian samples that had been stored for few months after processing to 13 for the Greek and 12 for the Iranian samples that had been stored for much longer periods of time. Despite these differences, six bands, 49.4 kDa, 38.5 kDa, 28.6 kDa, 25.3 kDa, 22.8 kDa and 19.5 kDa, were found to be common to all samples irrespective of processing or storage conditions.

Band at 49.4 kDa, identified as crocetin glucosyltransferase-2, as well as band at 38.5 kDa, identified as glyceraldhyde-3-phosphate dehydrogenase-2 are constantly present in high amount. Finally, band at 28.6 kDa was significantly less intense in the Italian sample compared to the other three samples. A comparison of the intensity for all bands is reported in Figure S4.

### 2.4. Diversity of Protein Pattern within Saffron Samples Originated from Italy

**Figure 4:**
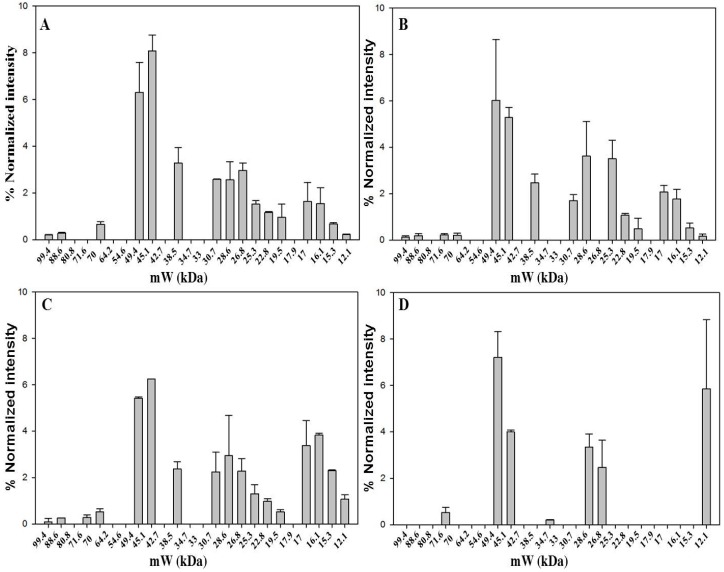
Bands detected in 1D-SDS-PAGE of dried saffron from Ramiseto (**A**); Itria (**B**); market (**C**) and Navelli (**D**). Gel image was acquired with ChemiDoc™ MP System imager and analyzed with Image Lab™ software (version 5.2). Data are from two replicates.

Four Italian saffron samples were analyzed by 1D-SDS-PAGE. Three samples are from different sites, Ramiseto in the Apennines between Tuscany and Emilia-Romagna, Itria a valley in the south of Italy, and Navelli (L’Aquila), one of the main sites of Italian saffron production in the central part of Italy. The latter sample is the only one that was received as a powder. The fourth sample was acquired in a market and was in stigma form. The 1D-SDS-PAGE analysis (Figure 4 and Figure S5) revealed 16 bands for Ramiseto and Itria samples, 17 for the commercial and only 7 for Navelli saffron. Both Ramiseto and Itria samples were about one year old, whereas Navelli sample was of unknown storage time. The low number of bands for Navelli saffron might indicate that the sample had been stored either much longer than one year after processing or under non-optimum conditions [27].

In all the Italian samples, bands at 49.4 kDa and 45.1 kDa accompanied by that at 28.6 kDa were the most relevant, in line with what was evidenced for saffron samples from different origins (see above). There is a very good similarity between the protein pattern of Ramiseto saffron and the commercial saffron, indicating that the latter is a pure, relatively fresh saffron. A detailed comparison of all bands for the fourth samples is reported in Figure S6.

### 2.5. Comparison of Protein Pattern of Saffron and Its Most Common Plant Adulterants

**Figure 5:**
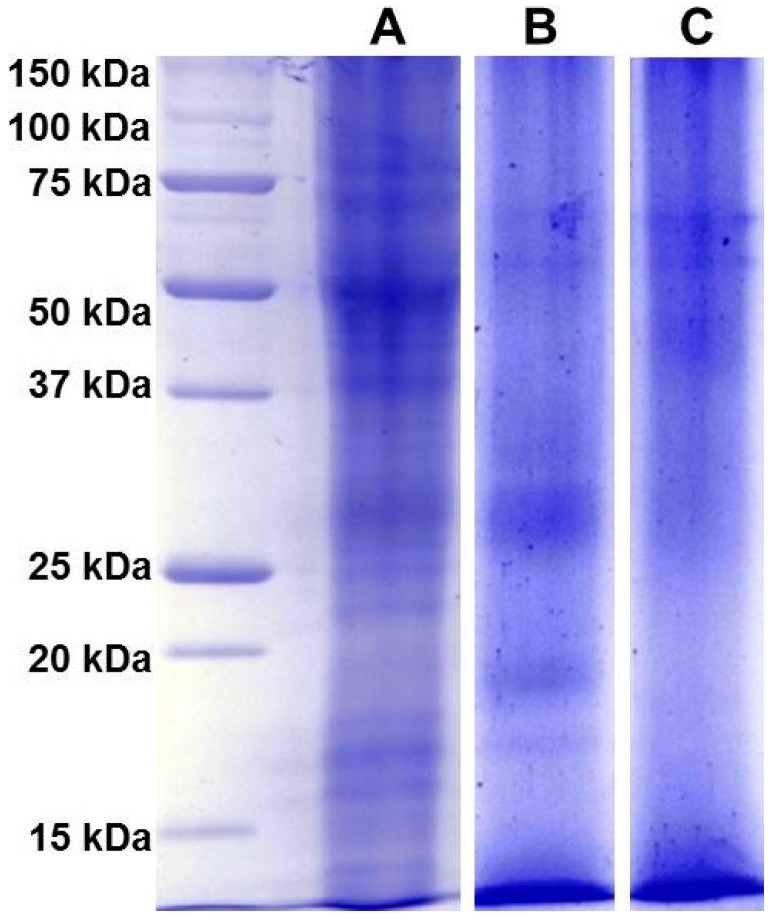
Proteins contained in dried Spanish saffron stigmas and styles (**A**), dried petals of *Carthamus tinctorius* L. (**B**) and dried fruits of *Gardenia jasminoides* (**C**) were separated by 1D-SDS-PAGE, and stained with Bio-safe™ Coomassie. The first lane at left contains molecular weight markers.

Saffron has a very high price, ranging from 12 to 20 Euro/g, depending on the quality category as defined by ISO3632-1 classes [33,34]. Therefore, not surprisingly, adulteration of saffron is a very common practice. Reported adulterants are dyes, flower stigmas or petals, colored meat, salts [17,18,19,20]. Petals of dried flowers of *Carthamus tinctorius* L. possess a dye, carthamin, that is a quinone-like compound, with coloring and flavoring reminiscent of saffron. Powder from the dried fruits of *Gardenia jasminoides* is also used for adulteration as they contain crocins, one of components of saffron. Dried petals of *Carthamus tinctorius* L. flowers and dried fruits of *Gardenia jasminoides* were used in this study as two of the most representative cases of saffron adulterants. The dried material from these plants was treated in the same way as saffron stigmas and styles in order to extract proteins (see Experimental Section). 1D-SDS-PAGE protein separation was then carried out as shown in Figure 5.

**Figure 6:**
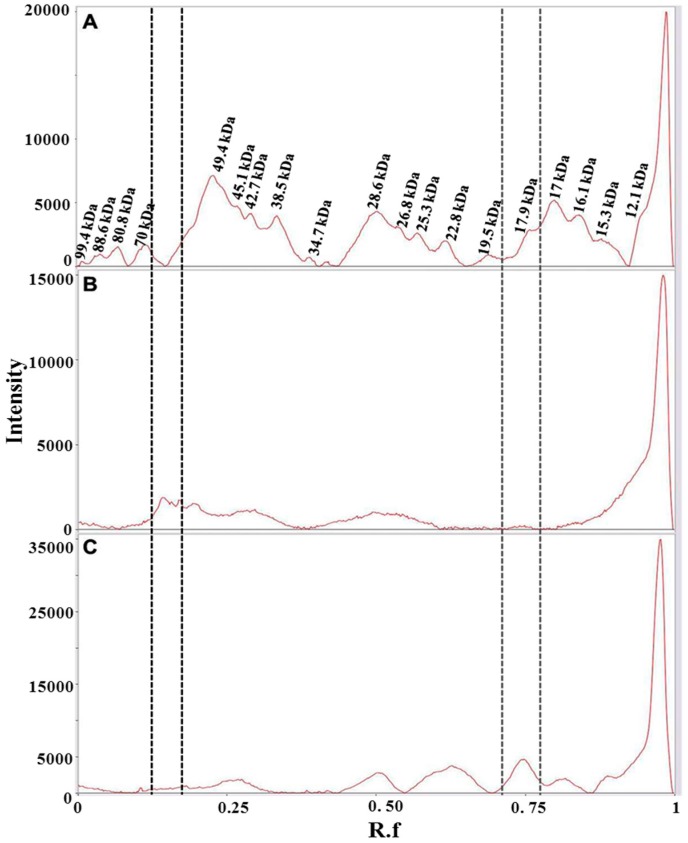
Bands detected in 1D-SDS-PAGE of two months aged dried stigmas and styles from Spain (**A**); dried petals of *Carthamus tinctorius* (**B**) and dried fruits of *Gardenia jasminoides* (**C**)*.* Gel image was acquired with ChemiDoc™ MP System imager and analyzed with Image Lab software (version 5.2). Vertical lines bracket bands that are uniquely present in either *Carthamus tinctorius* L. (black dotted lines) or *Gardenia jasminoides* (gray dotted lines)*.*

From the comparison of the protein profiles (Figure 6), obtained by gel imaging and analysis, it is evident that protein composition of the plant material from both *Carthamus tinctorius* and *Gardenia jasminoides* is very different from protein composition of saffron stigmas and styles. The bands at R_f_ of about 0.15 and 0.75 are most probably typical of *Carthamus tinctorius* L. petals and of *Gardenia jasminoides* fruits*,* respectively. Both bands were not detected in saffron. Taking into account that the amount of added adulterants in saffron is usually very high in order to be profitable (typically >5% *w*/*w*), our findings indicate the potential of a proteomic analysis in the detection of saffron adulteration with material from these plants.

## 3. Experimental Section

### 3.1. Saffron Samples

Saffron from different origins were obtained as follows. Three samples were offered by the “Azafran de La Mancha” Association (Camuñas, Toledo, Spain), one in the form of lyophilized fresh stigmas and styles while the others in the form of dried stigmas and styles that had been stored for two months and three years after processing. One sample, obtained from the French company Thiercelin (Paris, France), was Iranian saffron in stigma form, stored for about three years. Finally, one sample was offered by the Greek Saffron Producers Cooperative in Kozani (Krokos, Greece) in powder form and had been stored for about three years after harvest and processing. Three samples were offered by Italian producers and one obtained in a market from the Italian company Cannamela (Bologna, Italy). *Carthamus tinctorius* L. and *Gardenia jasminoides* plant materials were selected as potential adulterants and obtained from the sample collection of LFCT, University of Thessaloniki (Thessaloniki, Greece), as dried material. When received, samples were stored in the dark until the subsequent analysis.

### 3.2. Protein Extraction

The protein extraction procedure was designed in order first to remove as much as possible of colored metabolites that are present at high levels in the stigmas and styles of *C. sativus* L., and, subsequently, to extract protein components. Briefly, 100 mg of saffron stigmas and styles, corresponding to 10 flowers, were grounded to a fine powder. 5 mg were sonicated for 20 min with an ultrasonic bath in a solution of 10% TCA 0.07% (*w*/*v*) dithiothreitol (DTT) to extract metabolites. The solution was centrifuged for 15 min at 16.100 g and the supernatant was discharged. Two washes with chilled 0.07% DTT in 100% acetone were carried out. The sample pellet was suspended in 800 µL of a 25 mM Tris solution with 1% SDS (pH 7.4). Five cycles of sonication (30 s) were carried out with a low power (25%) using an ultrasonic device Q500 (Qsonica, Newtown, CT, USA) in order to achieve protein solubilization. The samples were centrifuged for 15 min at 16.100 *g*, the supernatant was recovered, and the protein fraction was isolated by overnight acetone ice cold precipitation. Finally, the protein component was suspended in the 25 mM Tris solution with 1% SDS (pH 7.4). The extraction procedure was carried out in triplicate for one of the samples, resulting in an average biological variation coefficient for the detected bands of 16.17%.

### 3.3. 1D-SDS-PAGE

SDS-PAGE was carried out using home-made polyacrylamide gels with 4% T% stacking and 12% T% resolving gels, in Tris-Glycine buffer system and 0.1% SDS. A volume corresponding to 5 µg of protein was added to sample buffer, boiled for 5 min and then separated at room temperature, applying 50 V for 30 min and 150 V until the end. Each sample was loaded in two separated lanes. After running, gels were incubated for 30 min with 20% ethanol, 10% acetic acid solution, washed in water for 10 min and proteins visualized by staining with Bio-safe™ Coomassie (Bio-Rad, Hercules, CA, USA). Unstained molecular weight markers from Bio-Rad were used as reference. Each sample was analyzed in duplicate. The gel image was acquired with ChemiDoc™ MP System imager (Bio-Rad). Gel analysis was performed with Image Lab software (version 5.2, Bio-Rad).

### 3.4. Image Analysis

SDS-PAGE gels were analyzed using Image Lab™ software (version 5.2, Bio-Rad). Software auto analysis procedure was applied to detect lanes and bands. Particularly, the band detection step was performed using the advanced option and the parameters set in the analysis are reported in Table 2.

Particularly, sensitivity determines the minimum optical density value of a band, the value size scale discriminates random fluctuations from the actual intensity value, signal noise filter reduces the noise in the gel image and, finally, the parameter shoulder allows to distinguish shoulders as separate bands. A further step was carried out by visually analyzing the electropherogram and gel image to confirm each detected band. Finally, only bands identified in both replicate lanes were included. Bands were normalized according to Image Lab band % parameter that is defined as the percentage of the band volume compared to all band volumes in each lane.

**Table 2:** Parameters used in Image Labs software for band detection step.

Parameter	Value
Sensitivity	50.00
Size Scale	5
Noise Filter	4
Shoulder	1

### 3.5. In Gel Digestion

In-gel digestion was performed as reported by Shevchenko and colleagues [35] with modifications. Briefly, gel bands were excised from SDS-PAGE and completely de-stained with 20% ethanol, 10% acetic acid solution. A wash in 25 mM ammonium bicarbonate was carried out to eliminate all the de-staining solution. Subsequently, the gel bands were reduced with 1% DTT in 25 mM ammonium bicarbonate for 15 min and alkylated with 4% iodoacetamide in 25 mM ammonium bicarbonate for 15 min in the dark. Two washes of 20 min were carried out with 25 mM ammonium bicarbonate–100% acetonitrile (50:50). A further wash was performed for 5 min with 100% acetonitrile. The bands were completely dehydrated with a speed vacuum centrifuge (Christ, Osterode am Harz, Germany) and rehydrated with 50 µL for 15 min at 25 °C with a solution 10 µg/mL Proteomic grade porcine trypsin (Sigma-Aldrich, Saint Louis, MO, USA) in 20 mM ammonium bicarbonate, 9% *v*/*v* acetonitrile and 0.1 mM hydrochloric acid. Gel bands were incubated for 16 h at 37 °C in 25 mM ammonium bicarbonate solution. Finally, peptides were eluted from gel bands with 100%–0.1% trifluoroacetic acid 50:50.

### 3.6. Mass Spectrometry

Peptide mass fingerprinting was carried out with 4800 Plus MALDI TOF/TOF™ AB SCIEX. A 10 mg/mL α-Cyano-4-hydroxycinnamic acid (α-CHCA) MALDI matrix was prepared by dissolving α-CHCA (Sigma-Aldrich) in 100% Acetonitrile–0.1% TFA 1:1. The MALDI spot were prepared by mixing one µL of protein digest sample with one µL of α-CHCA solution. One µL of peptide-matrix mixture was spotted on the MALDI plate. The instrument was operated in positive mode and acquisition mass range was set to 600–4000 Da. Typically, for each spot 500 shots were combined. Mmass 5.5 open source software [36] was used to create a peak list for each spot that were manually checked for the presence of signal from matrix complex, trypsin and human keratin peptides. The resulting peptide mass lists were submitted to database search using Mascot Server (Matrix Science) engine against Swiss Prot *Viridiplantae* database. The peptide mass tolerance was set at 100 ppm, two missing cleavages of trypsin were tolerated and carbamidomethylation was set as fixed modification while methionine oxidation was considered an optional modification.

## 4. Conclusions

This is the first investigation about the exploitation of proteomic tools for the characterization of saffron that could be also applied to reveal possible fraud with common plant adulterants*.* The so far findings indicate that (i) few bands are present in all saffron samples independently of origin and storage time, with amounts that significantly vary among samples; (ii) aging during saffron storage is associated with a reduction of the number of detectable bands, suggesting that proteases are still active; (iii) the protein pattern of saffron is quite distinct from those of two common adulterants, such as the dried petals of *Carthamus tinctorius* and the dried fruits of *Gardenia jasminoides.* Overall, this study represents a step towards the development of a proteomic database containing the profile and identity of saffron proteins as well as the protein profile of common plant adulterants.

## Supplementary Materials

Supplementary materials can be accessed at: http://www.mdpi.com/1420-3049/21/2/167/s1.
