# Supplementary material for: Insight of Saffron Proteome by Gel-Electrophoresis
Source: Molecules. 2016 Jan 29;21(2):167. doi: 10.3390/molecules21020167 (PMC6273178; doi:10.3390/molecules21020167)
Supplement: Supplementary file 1 [file molecules-21-00167-s001.pdf]

Supplementary Materials: Insight of Saffron Proteome by Gel-Electrophoresis

Gianluca Paredi, Samanta Raboni, Francesco Marchesani, Stella A. Ordoudi, Maria Z. Tsimidou and Andrea Mozzarelli

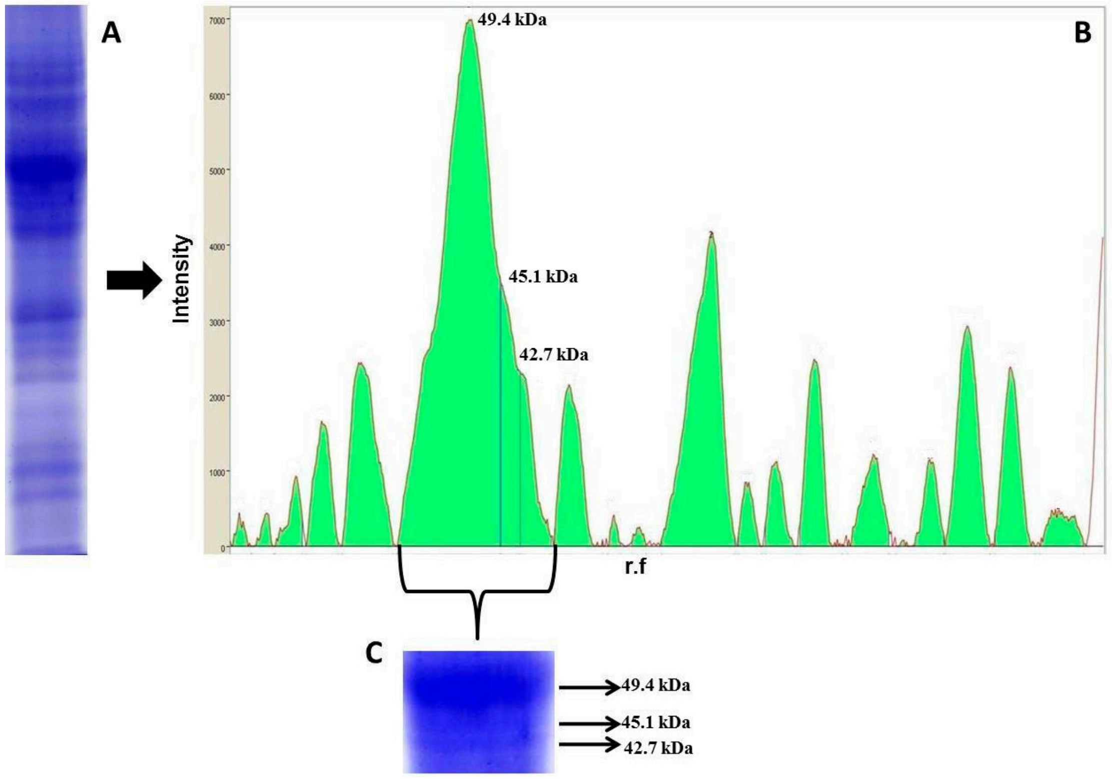

Figure S1. Gel image analysis. Panel **A**: gel lane from Fresh Spanish saffron Panel **B**: Electropherogram; Panel **C**: Enlargement of gel region containing bands 6, 7 and 8.

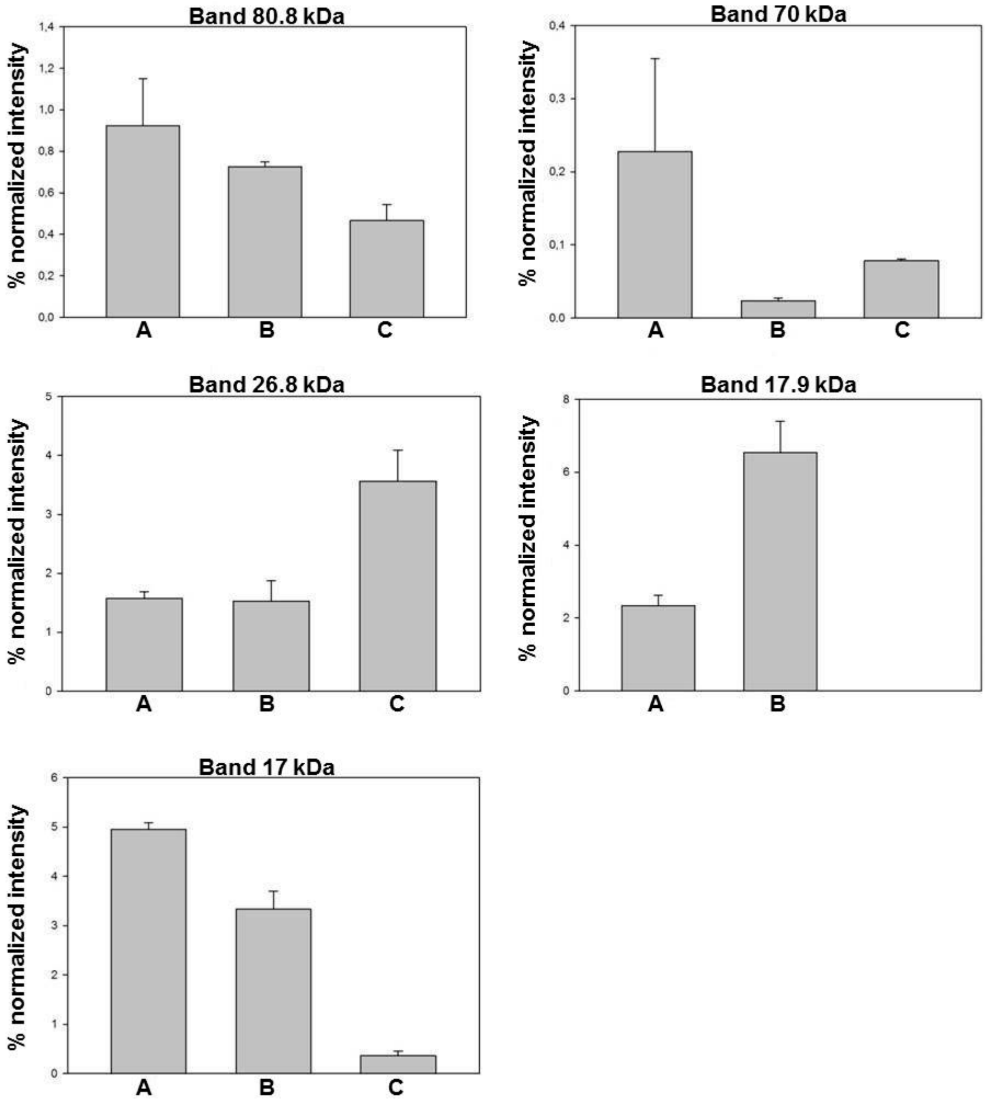

Figure S2. Relative band intensity of selected bands for Spanish saffron fresh stigmas and styles (**A**); dried stigmas and styles stored for two months (**B**); and dried stigmas and styles stored for three years (**C**).

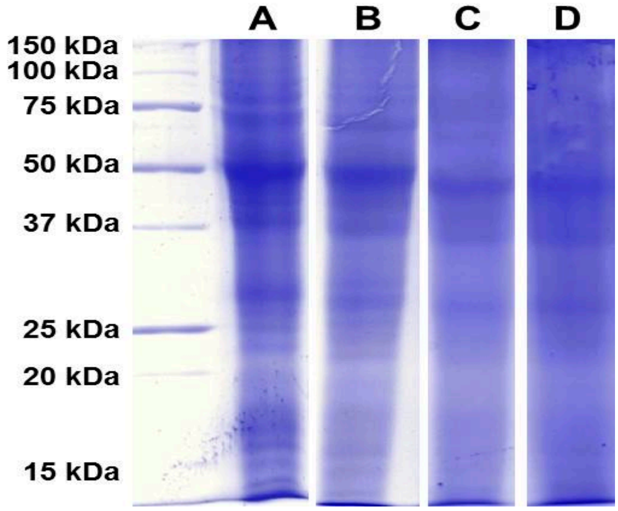

Figure S3. The proteins contained in saffron dried stigmas and styles from Spain (**A**); Italy (**B**); Greece (**C**) and Iran (**D**) were separated by 1D-SDS-PAGE and stained with Biosafe Coomassie. The first lane at left contains molecular weight markers.

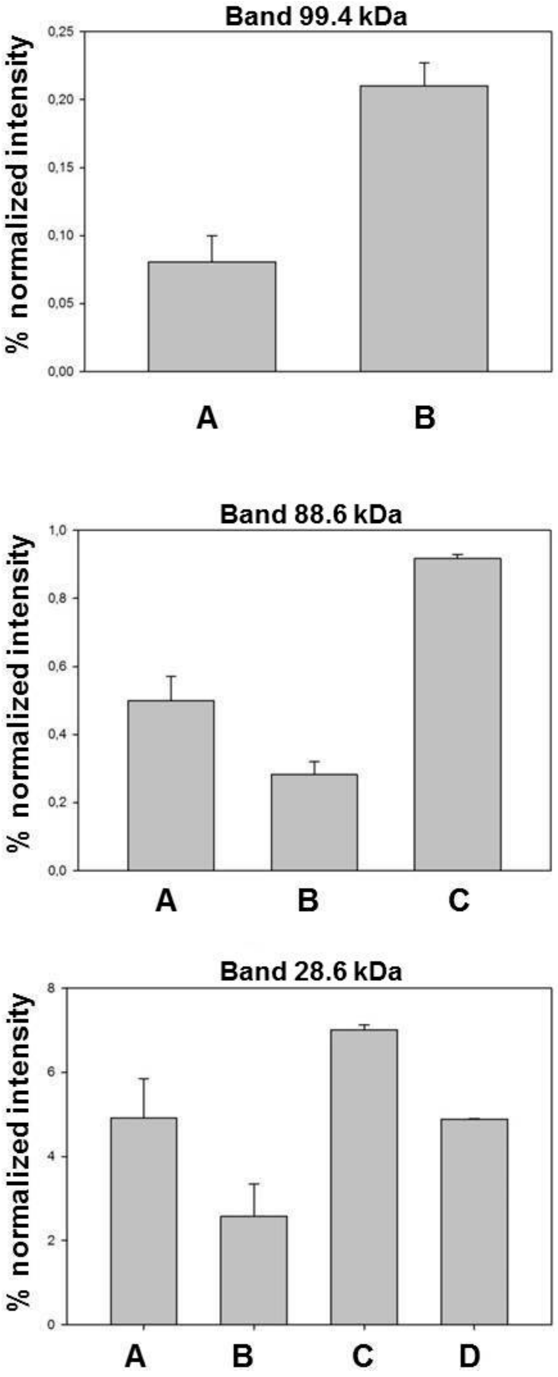

Figure S4. Relative band intensity of selected bands for saffron dried stigmas and styles from Spain (**A**); Italy (**B**); Greece (**C**) and Iran (**D**).

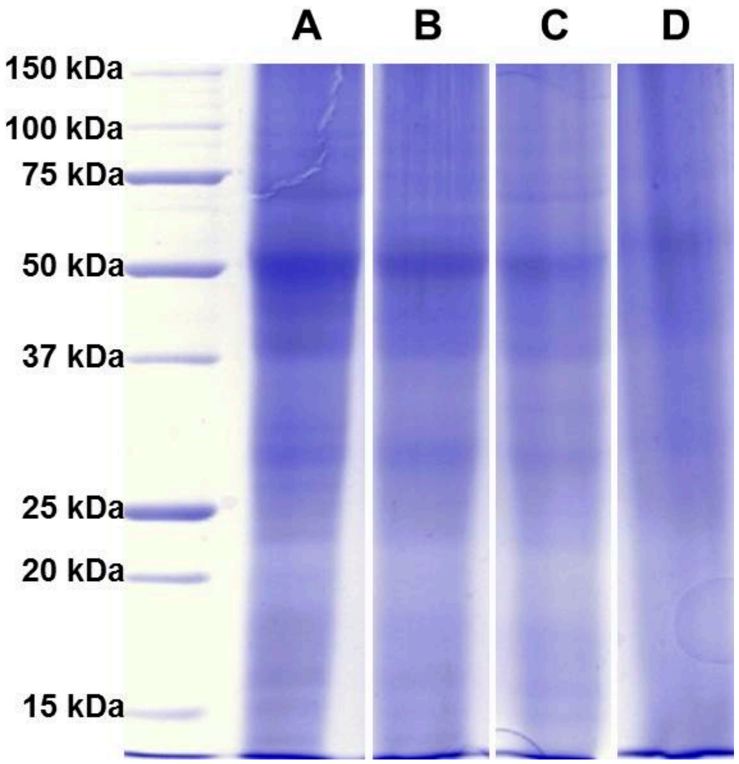

Figure S5. The proteins contained in saffron dried stigmas and styles from Ramiseto (**A**); Itria (**B**); market (**C**) and Navelli (**D**) were separated by 1D-SDS-PAGE, and stained with BiosafeCoomassie. The first lane at left contains molecular weight markers.

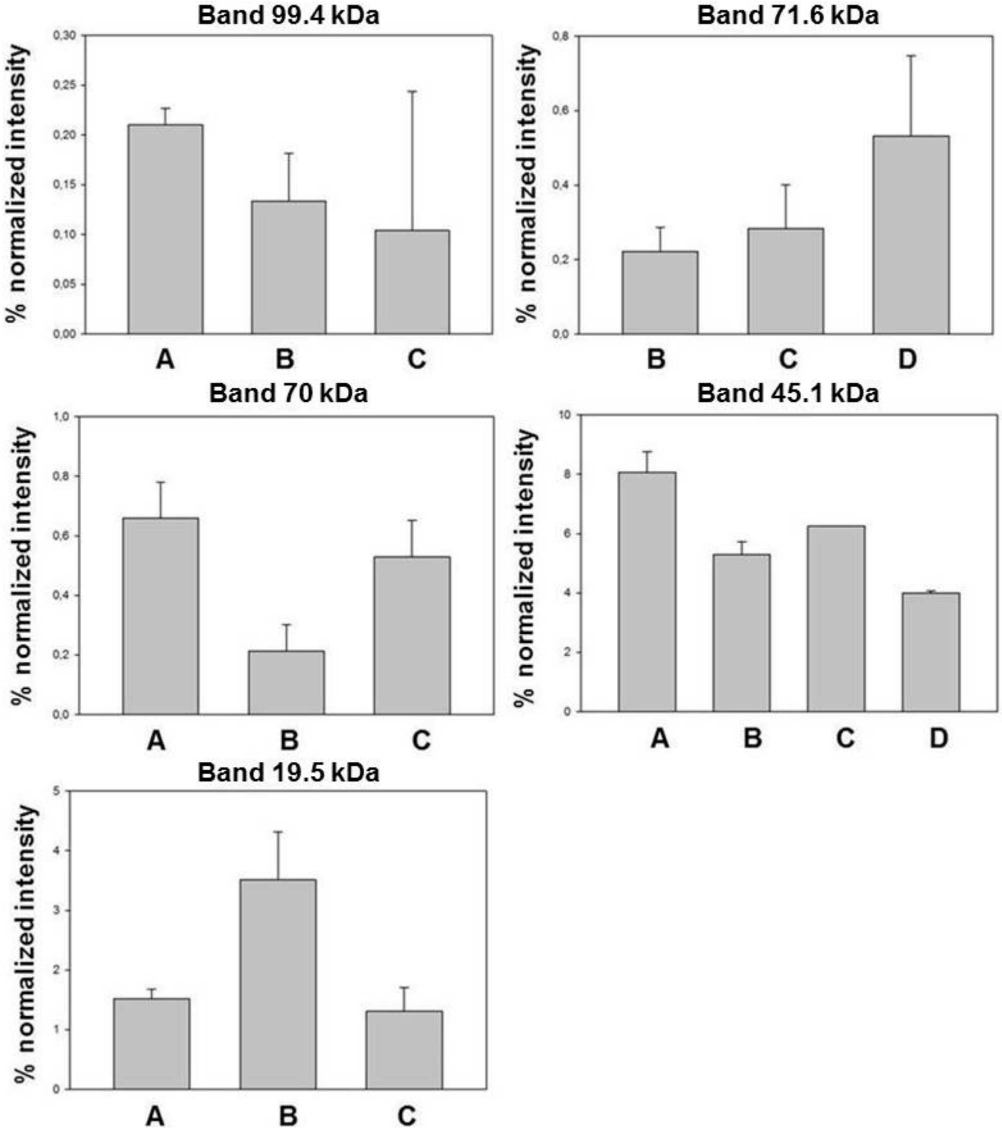

Figure S6. Relative band intensity of selected bands for saffron dried stigmas and styles from Ramiseto (**A**); Itria (**B**); market (**C**) and Navelli (**D**).

Table S1. Bands detected in the analyzed saffron samples with corresponding molecular weights.

MW(kDa)	Spanish Fresh	Spanish Dried	Spanish Stored	Ramiseto	Itria	Italian Commercial	Aquila	Greece	Iran
99.4	X	X		X	X	X			
88.6	X	X		X	X	X		X	
80.8	X	X	X					X	X
71.6					X	X	X		
70	X	X	X	X	X	X		X	
64.2	X							X	X
54.6	X								X
49.4	X	X	X	X	X	X	X	X	X
45.1	X	X		X	X	X	X		
42.7	X	X	X					X	X
38.5	X	X	X	X	X	X		X	X
34.7	X	X					X		
33	X					X			
30.7				X	X	X			
28.6	X	X	X	X	X	X	X	X	X
26.8	X	X	X	X		X	X		
25.3	X	X		X	X	X		X	X
22.8	X	X		X	X	X		X	X
19.5	X	X	X	X	X	X		X	X
17.9	X	X							
17	X	X	X	X	X	X			X
16.1	X	X		X	X	X		X	
15.3	X	X		X	X	X		X	
12.1	X	X		X	X	X	X		X

Table S2. Amino acid sequences of identified peptides.

Protein	Peptide
Phospoenolpyruvate carboxylase 3	R.NIEKMASIDAQLR.Q<br>K.VSEDDKLVEYDALLLDR.F<br>K.LVEYDALLLDR.F<br>R.QVSTFGLSLVR.L<br>R.QEWLLAELSGK.R<br>K.RPLFGPDLPK.T<br>R.LFSIDWYKNRINGK.Q<br>K.AQEELVK.V<br>R.SVVFQEPR.F<br>R.FVEYFR.L<br>R.LATPELEYGR.M<br>K.RKPSGGIESLR.A<br>R.AIPWIFAWTQTR.F
Heat shock cognate 70 kDa protein	R.VEIIANDQGNR.T<br>K.NAVVTVPAYFNDSQR.Q<br>R.IINEPTAAAIAYGLDKK.A<br>K.ATAGDTHLGGEDFDNR.M<br>R.MVNHFVQEFK.R + Oxidation (M)<br>K.KDISGNPR.A<br>R.TLSSTAQTTIEIDSLYEGIDFYSTITR.A<br>R.ARFEELNMDLFR.K<br>R.ARFEELNMDLFR.K + Oxidation (M)<br>R.FEELNMDLFR.K<br>R.FEELNMDLFR.K + Oxidation (M)<br>K.CLRDAKMDK.S<br>K.VQQLLQDFFNGK.E<br>K.EQVFSTYSDNQPGVLIQVYEGER.A<br>K.ITITNDKGR.L<br>K.SEDEELKKK.V<br>K.NALENYAYNMR.N
Crocetin glucosyltransferase-2	R.LASHNLLTTLVNTR.F<br>R.SVAFFTQPCAVDTIYR.H<br>R.HVWEGR.I<br>R.IKVPVAEPVR.L<br>K.NLDKADMMGR.N + Oxidation (M)<br>K.ADMMGR.N + 2 Oxidation (M)<br>K.SIGPTVPSTYLDNR.I<br>K.SFIWVVR.T<br>K.LPANFTQENASR.G<br>K.YVEDVWK.V<br>K.YVEDVWKVGVR.A<br>K.VGVRAKTYGK.D<br>R.GEEFKR.C<br>R.CVEEVMDGERSGKIR.E<br>K.EFIHQCCNDSKISLV.

Table S2. *Cont.*

Alpha-1,4 glucan-proteinsynthase	K.VIKVPEGFDYELYNR.N<br>K.VIKVPEGFDYELYNRNDINR.I<br>K.VPEGFDYELYNR.N<br>K.VPEGFDYELYNRNDINR.I<br>K.NLLSPSTPFFFNTLYDPYR.E<br>K.NLLSPSTPFFFNTLYDPYREGTDFVR.G<br>R.GYPFSLR.E<br>R.ELIGPAMYFGLMGDGQPIGR.Y<br>R.ELIGPAMYFGLMGDGQPIGR.Y + Oxidation (M)<br>R.ELIGPAMYFGLMGDGQPIGR.Y + 2 Oxidation (M)<br>K.TGLPYIWHSK.A
Glycerladehyde-3-phosphate dehydrogenase-2	M.AKIKIGINGFGR.I<br>K.IKIGINGFGR.I<br>K.YDTVHGQWK.H<br>K.HHEVKVK.D<br>K.EVTVFGCR.N<br>K.AAAHLKGGAK.K<br>K.DAPMFVVGVNEK.E + Oxidation (M)<br>K.TVDGPSSKDWR.G<br>R.VPTVDVSVVDLTVR.L<br>K.AAIKEESEGK.L<br>K.AGIALNDNFVK.L<br>R.VVDLIR.H<br>R.VVDLIRHMYNTQ.
